# Endoscopic image classification algorithm based on Poolformer

**DOI:** 10.3389/fnins.2023.1273686

**Published:** 2023-09-21

**Authors:** Huiqian Wang, Kun Wang, Tian Yan, Hekai Zhou, Enling Cao, Yi Lu, Yuanfa Wang, Jiasai Luo, Yu Pang

**Affiliations:** ^1^Postdoctoral Research Station, Chongqing Key Laboratory of Photoelectronic Information Sensing and Transmitting Technology, Chongqing University of Posts and Telecommunications, Chongqing, China; ^2^Chongqing Xishan Science & Technology Co., Ltd., Chongqing, China

**Keywords:** endoscopic image, image classification, Poolformer, token mixer, ConvNeXt, single-path topology only during inference

## Abstract

Image desmoking is a significant aspect of endoscopic image processing, effectively mitigating visual field obstructions without the need for additional surgical interventions. However, current smoke removal techniques tend to apply comprehensive video enhancement to all frames, encompassing both smoke-free and smoke-affected images, which not only escalates computational costs but also introduces potential noise during the enhancement of smoke-free images. In response to this challenge, this paper introduces an approach for classifying images that contain surgical smoke within endoscopic scenes. This classification method provides crucial target frame information for enhancing surgical smoke removal, improving the scientific robustness, and enhancing the real-time processing capabilities of image-based smoke removal method. The proposed endoscopic smoke image classification algorithm based on the improved Poolformer model, augments the model’s capacity for endoscopic image feature extraction. This enhancement is achieved by transforming the Token Mixer within the encoder into a multi-branch structure akin to ConvNeXt, a pure convolutional neural network. Moreover, the conversion to a single-path topology during the prediction phase elevates processing speed. Experiments use the endoscopic dataset sourced from the Hamlyn Centre Laparoscopic/Endoscopic Video Dataset, augmented by Blender software rendering. The dataset comprises 3,800 training images and 1,200 test images, distributed in a 4:1 ratio of smoke-free to smoke-containing images. The outcomes affirm the superior performance of this paper’s approach across multiple parameters. Comparative assessments against existing models, such as mobilenet_v3, efficientnet_b7, and ViT-B/16, substantiate that the proposed method excels in accuracy, sensitivity, and inference speed. Notably, when contrasted with the Poolformer_s12 network, the proposed method achieves a 2.3% enhancement in accuracy, an 8.2% boost in sensitivity, while incurring a mere 6.4 frames per second reduction in processing speed, maintaining 87 frames per second. The results authenticate the improved performance of the refined Poolformer model in endoscopic smoke image classification tasks. This advancement presents a lightweight yet effective solution for the automatic detection of smoke-containing images in endoscopy. This approach strikes a balance between the accuracy and real-time processing requirements of endoscopic image analysis, offering valuable insights for targeted desmoking process.

## 1. Introduction

Endoscopes are essential tools that utilize the body’s natural cavities or tiny incisions to provide real-time visualization of internal organs and tissues (Fu et al., 2021; Boese et al., 2022; Chadebecq et al., 2023). This minimizes the need for larger incisions during surgery, leading to shorter patient recovery periods. Consequently, endoscopy is now extensively employed in examining and treating various diseases involving the gastrointestinal tract (Aceves et al., 2022; Niknam et al., 2022), ear, nose, throat (Bastier et al., 2022; Poutoglidis et al., 2022), spine (Ahn, 2020; Simpson et al., 2022) and urinary system(Zou et al., 2020; Yamashita et al., 2022). Despite the benefits of endoscopy, challenges arise during procedures: the generation of smoke due to the destruction and vaporization of tissue proteins and fat by the instruments (Yi et al., 2023). This smoke hinders the visibility of tissue structures in endoscopic images, obstructing the physician’s vision and impeding accurate judgment and treatment. To address this challenge, image-based surgical smoke analysis and processing have emerged as a promising solution. Not constrained by hardware limitations, this approach reduces the reliance on surgical aids and assists physicians in obtaining clearer views for more precise diagnoses and treatments. Consequently, it holds immense potential and value for clinical applications.

However, the existing methods for intelligent analysis and processing of surgical smoke primarily focus on desmoking endoscopic images. For instance, Wang et al. (2019) proposed an improved convolutional neural network (CNN) with an encoder-decoder architecture for real-time surgical smoke removal. Their network takes an image with smoke along with its laplacian image pyramid decomposition as input and produces an image with smoke removed. To create the synthetic dataset, they utilized Blender and Adobe Photoshop to add rendered smoke to clean images. Similarly, Lin et al. (2021) introduced a supervised UNet-based network where the Laplace pyramid is fused at the encoder, and the CBAM module is integrated at the decoder. They employed Blender to generate datasets of laparoscopic images with varying levels of light and dense smoke. Their method achieved a high structural similarity of 0.945 and a peak signal-to-noise ratio of 29.27 for the test data. Furthermore, Bolkar et al. (2018) constructed a synthetic surgical desmoking dataset. They adapted the integrated desmoking network, AOD-Net, initially designed for outdoor desmoking, and their proposed supervisory model comprises five convolutional layers with ReLU activation units and three cascade layers. Azam et al. (2022) removed smoke from laparoscopic images by manual multiple exposure image fusion method. Venkatesh et al. (2020), Pan et al. (2022), Zhou et al. (2022), and Su and Wu (2023) respectively used CycleGAN-based network structure to realize laparoscopic image de-smoking and affirmed the important role of smoke detection in laparoscopic image desmoking, but their main design focus was on the structure of smoke purification network. Additionally, Wang et al. (2023) proposed a desmoking method based on Swin transformer, employing Swin transformer blocks to extract deep features. Most of the aforementioned desmoking techniques process all endoscopic images within the video stream for smoke removal, which is inefficient because smoke is not consistently present throughout the entire surgical procedure, and a substantial portion of the video stream consists of smoke-free images. Processing all video stream images for de-smoking not only increases computational demands but may also introduce new noise into the original smoke-free images. Hence, it becomes imperative to differentiate between smoked and smoke-free images, enabling the smoke cleaning algorithms to selectively focus on desmoking only the images containing smoke, while leaving the smoke-free images unaltered. This targeted approach ensures more efficient and precise desmoking, preserving the clarity and integrity of the original smoke-free images. This approach would significantly reduce equipment resource requirements, improve processing speed, and enhance the real-time, accuracy, and scientificity of desmoking in endoscopic scenarios.

To date, few studies specifically focus on the classification of endoscopic images containing smoke. Nevertheless, endoscopic image classification aligns with the fundamental principles of other image binary/multi-classification problems, wherein the objective is to predict input images into multiple categories based on their distinctive features. In the early stages, researchers employed algorithms like k-nearest neighbors, Support Vector Machine, and Random Forest for such tasks. These methods typically involved feature extraction prior to classification, necessitating human selection of one or more features that influenced the classification quality. In recent years, CNNs have gained prevalence for image classification due to their ability to automatically extract relevant image features and demonstrate exceptional performance on large-scale datasets. Lecun et al. (1998) proposed an early CNN architecture, comprising two convolutional layers, two pooling layers, and three fully connected layers, which facilitated the classification and recognition of handwritten digits and laid the groundwork for subsequent image classification models. Notably, Krizhevsky et al. (2012) introduced AlexNet, which achieved groundbreaking results in the ImageNet image classification competition. Their work significantly improved performance on large-scale image datasets. Additionally, Tan and Le (2019) introduced EfficientNet, a CNN structure optimized through neural network search technology. Furthermore, ResNet was proposed as an innovative deep residual learning framework to address the issue of gradient explosion in deep network training (He et al., 2016). Howard et al. (2017) proposed MobileNet, a lightweight deep neural network designed for embedded devices. MobileNet utilizes depth-wise separable convolution to efficiently reduce the number of model operations and parameters, making it well-suited for resource-constrained environments. Dosovitskiy et al. (2021) made a significant breakthrough in image classification by directly applying the transformer architecture to this domain, introducing the vision transformer (ViT) model. The ViT model utilizes the transformer’s encoder to extract essential features from images, resulting in remarkable advancements in image classification. In a related development, Yu et al. (2022) proposed the Poolformer model. Instead of employing the attention module, the Poolformer model utilizes a straightforward spatial pooling operation. Even with fewer parameters, the Poolformer model achieves competitive performance in image classification tasks. Furthermore, Almeida et al. (2022), Dewangan et al. (2022), and Zhao et al. (2022) individually explored lightweight CNNs for smoke detection in images of natural scenes.

Among existing image classification methods, network models like Poolformer have demonstrated the capability to achieve highly accurate real-time recognition in natural images. They hold significant potential for extending their effectiveness to the detection of endoscopic smoke-containing images. However, compared to natural images, endoscopic images face distinctive challenges in feature extraction and recognition. This is primarily due to the non-Lambertian reflective properties of human tissues, resulting in weak texture features and a lack of salient features. Furthermore, the classification of endoscopic smoke-containing images necessitates real-time performance during surgical procedures, where achieving a high level of real-time efficiency is critical for successful implementation. The characteristics inherent in endoscopic scenes introduce complexity to the task of automatic feature extraction and recognition.

To enhance real-time performance while maintaining accuracy in smoke detection on endoscopic images with weak textures, this paper proposes a method for endoscopic smoke image classification using Poolformer. The primary enhancement of the algorithm lies in the model’s encoder, where the Token Mixer is upgraded from a basic pooling layer to a multiplexed branching structure akin to the purely convolutional neural network ConvNeXt (Liu et al., 2022). During prediction, it is further transformed into a single-path topology to bolster the model’s inference speed.

## 2. The proposed method

### 2.1. Overview

**Figure 1:**
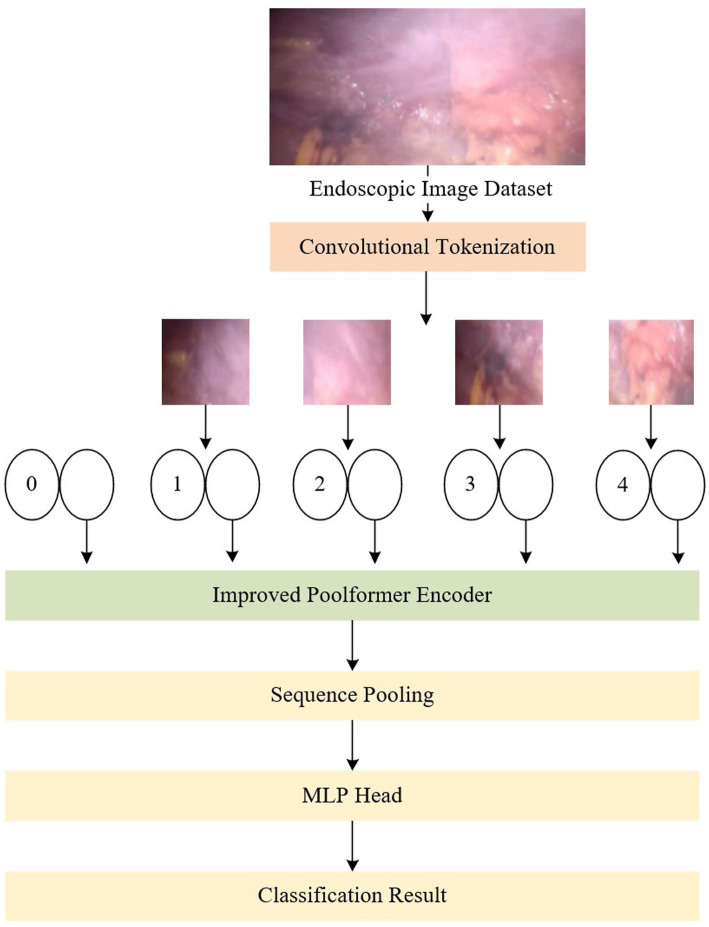
An overview of the proposed network, which consists of convolution module, improved Poolformer encoder, sequence pooling and MLP head.

The Poolformer-based network for endoscopic image classification proposed in this paper is depicted in Figure 1. In terms of the network structure, the original Poolformer replaces the Multi-head Attention module in the encoder block of the conventional vision transformer with a simple pooling layer. To further enhance the feature extraction capabilities for weakly textured images, this paper proposes the design of a multi-branch pure convolutional neural network structure similar to ConvNeXt, aiming to optimize the pooling layer in the original Poolformer model. This enhancement improves the model’s feature extraction ability. Furthermore, to ensure real-time processing in endoscopic video streaming, the model’s structure is transformed into a one-way model to obtain classification results through predictive reasoning during the testing process.

### 2.2. Convolution module

In the Vision Transformer (ViT) module (Dosovitskiy et al., 2021), input tokens (vectors) are essential for processing images of various sizes. As an example, in the ViT-B/16 model, the input image, $x\in {\mathbb{R}}^{h\times w\times c}$, where *h* denotes the height, *w* signifies the width, and *c* represents the number of channels, undergoes convolution with a kernel size of 16 × 16, a stride of 16, and employs 768 convolution kernels to accomplish this operation. This process involves partitioning the input image *x* into patches of size 16 × 16. While increasing the convolutional kernel and step size in large datasets can expand the receptive field, allowing for feature maps over a wider area and obtaining superior global features, in smaller datasets, such as medical datasets like endoscopes, this advantage may lead to the loss of detailed information between patches.

**Figure 2:**
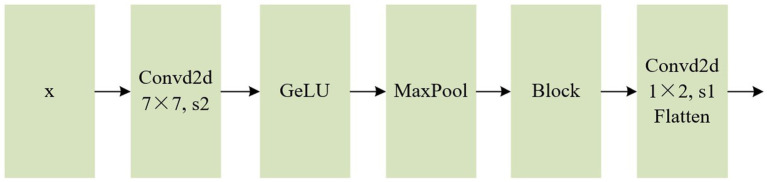
Flow chart of convolution module.

To tackle this issue, this paper adopts the convolution-based patching method, which effectively mitigates the loss of detailed information. This approach removes the constraint that each patch size must be a multiple of the image’s dimensions, enabling adaptation to datasets with varying size dimensions. As illustrated in Figure 2, the preprocessed input vector *x* undergoes feature extraction through convolution, activation function, and maximum pooling operations. A downsampling operation is applied to meet the input specifications of the subsequent Positional Embedding layer. The GELU activation function is integrated in order to introduce randomness by combining it with the concept of dropout, thereby enhancing the robustness of the model. Additionally, to address the degradation problem, a residual module based on ResNet (He et al., 2016) is employed. Finally, a positional embedding layer vector of the same size as ViT is obtained through a convolution and flattening operation.

### 2.3. Improved Poolformer encoder

**Figure 3:**
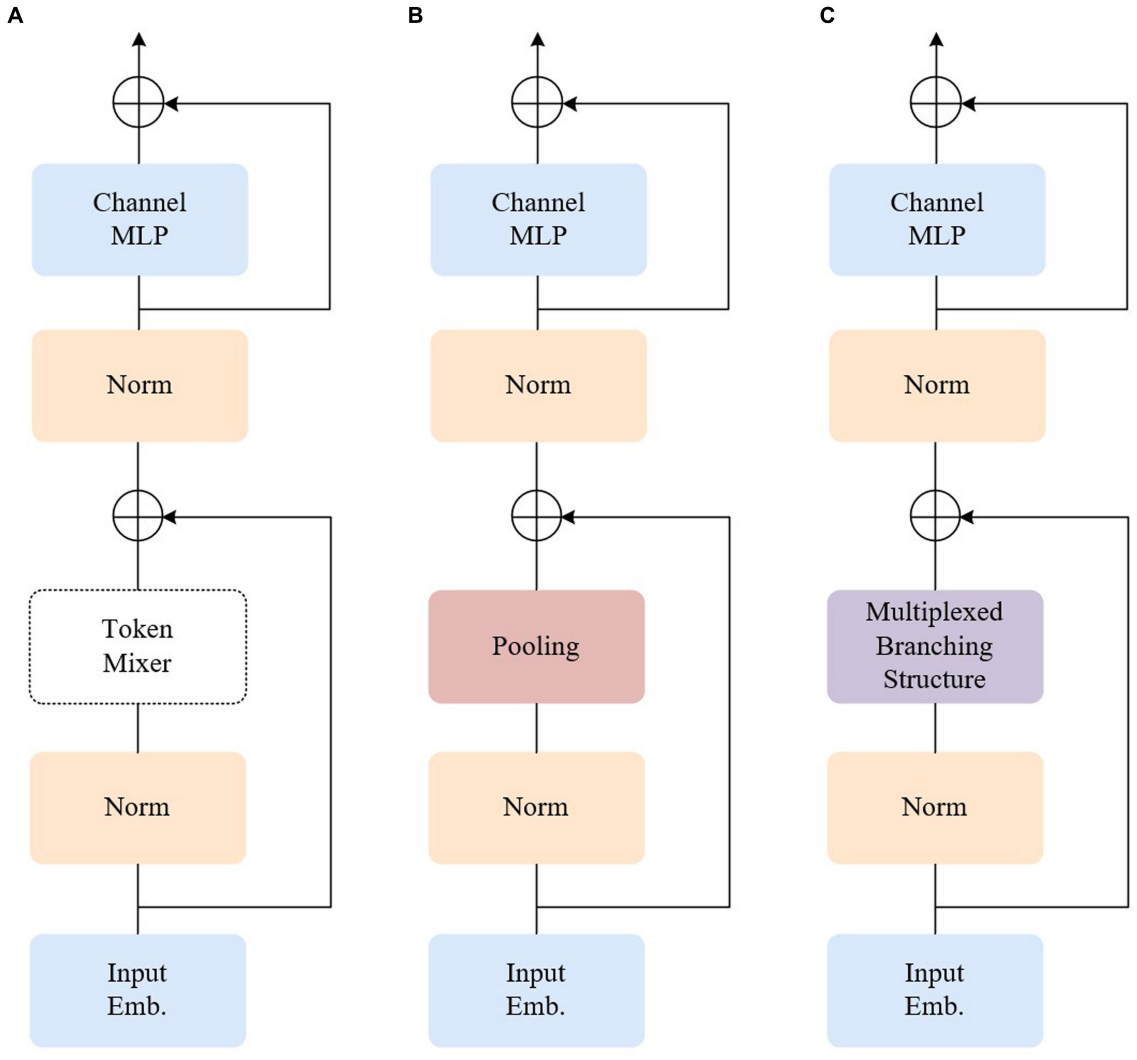
Illustrations of the architecture of different encoders. **(A)** MetaFormer. **(B)** Poolformer. **(C)** Our model.

The encoder of the fundamental ViT model primarily comprises two components: an attention module, also known as the token mixer, which facilitates information exchange between tokens, and subsequent elements such as channel MLP and residual connections. Abstracting the architecture while disregarding the specifics of how the token mixer is implemented with an attention module, the aforementioned design can be represented as the MetaFormer architecture (Yu et al., 2022), depicted in the first panel of Figure 3A. Contrasting with the conventional ViT model, the Poolformer model transforms the multi-head attention mechanism into a simple pool pooling layer, as illustrated in Figure 3B. Leveraging the overall superiority of the entire MetaFormer framework and the inclusion of the pooling layer, it significantly reduces the computation burden, machine load, and required video memory.

The pooling layer, in the process of dimensionality reduction, may lead to the loss of local information, which is particularly critical in weak texture endoscopic images where local information plays a crucial role. It is essential to minimize information loss as much as possible. Convolutional neural networks excel at retaining local information compared to pooling layers. Leveraging this advantage, the token mixer part is optimized to adopt a ConvNeXt-like multiplexed branching structure, as depicted in Figure 3C. ConvNeXt is a pure convolutional neural network architecture that competes with transformer networks. In comparison to the transformer model, ConvNeXt significantly reduces the number of parameters, introduces spatial inductive bias, and eliminates positional bias. Consequently, this acceleration of network convergence leads to a more stable network training process. Through modifications involving stage proportions, grouping convolutions, an anti-bottleneck design, utilization of larger convolutional kernels in finer details, and replacing the activation function, ConvNeXt achieves faster inference speed and higher accuracy than the Swin Transformer.

**Figure 4:**
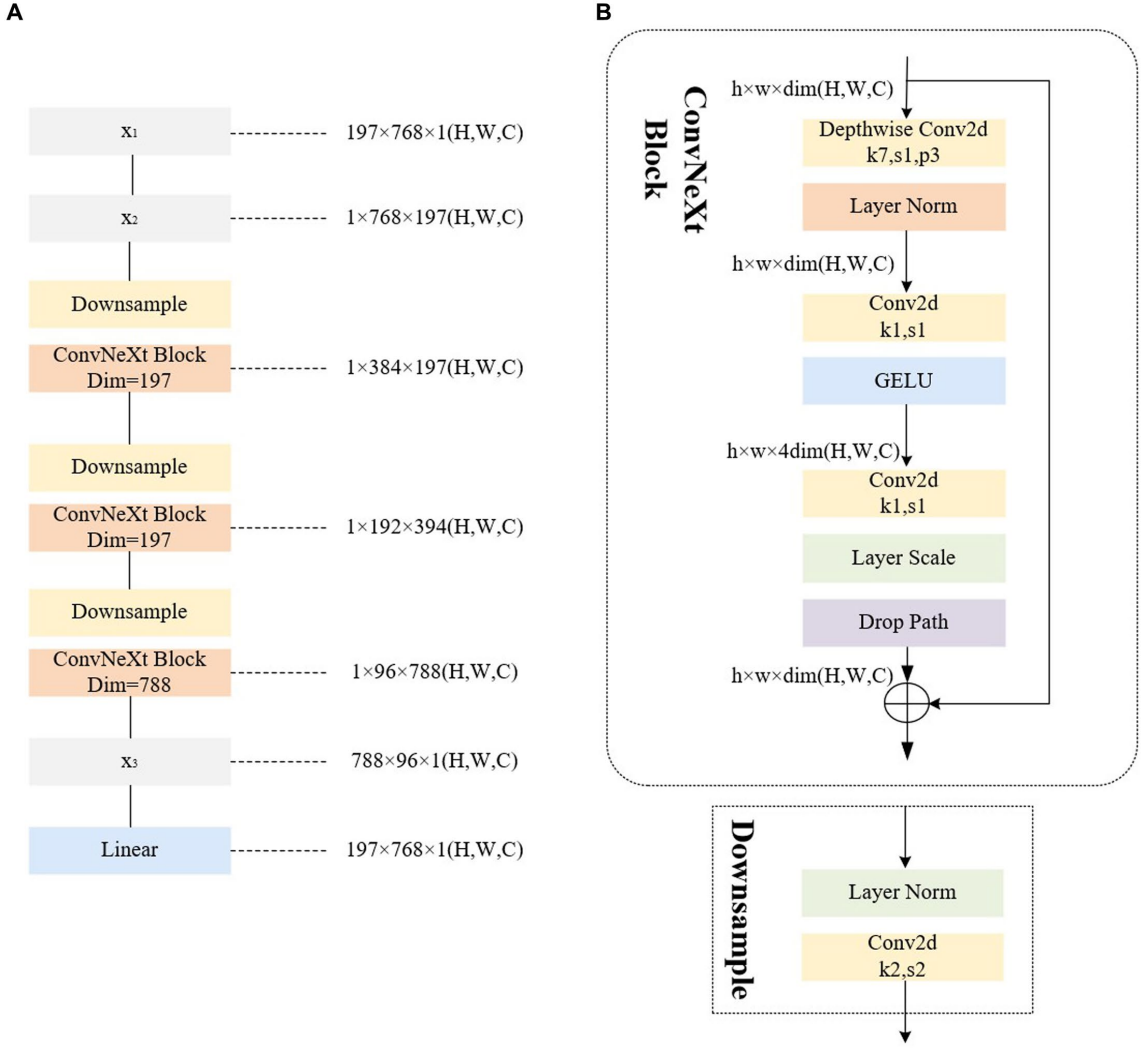
Illustrations of the architecture of our improved encoder. **(A)** The improved encoder. **(B)** The ConvNeXt block.

For the improved Poolformer encoder, the 2D matrix *x*_1_ obtained from the input image through the convolution operation and flattening operation in Figure 2 serves as the input sequence. The specific structure and steps, for example, using ViT-B/16 (where the 2D matrix *x*_1_ is in the format of [197,768]), are illustrated in Figure 4. In step (1), *x*_1_ undergoes mapping to interchange the *H* (height) dimension and *C* (channel) dimension, resulting in the matrix *x*_2_. A similar operation is performed in step (2), where the height dimension containing class categorization information is considered as the channel dimension.

### 2.4. RepConvNeXt block

**Figure 5:**
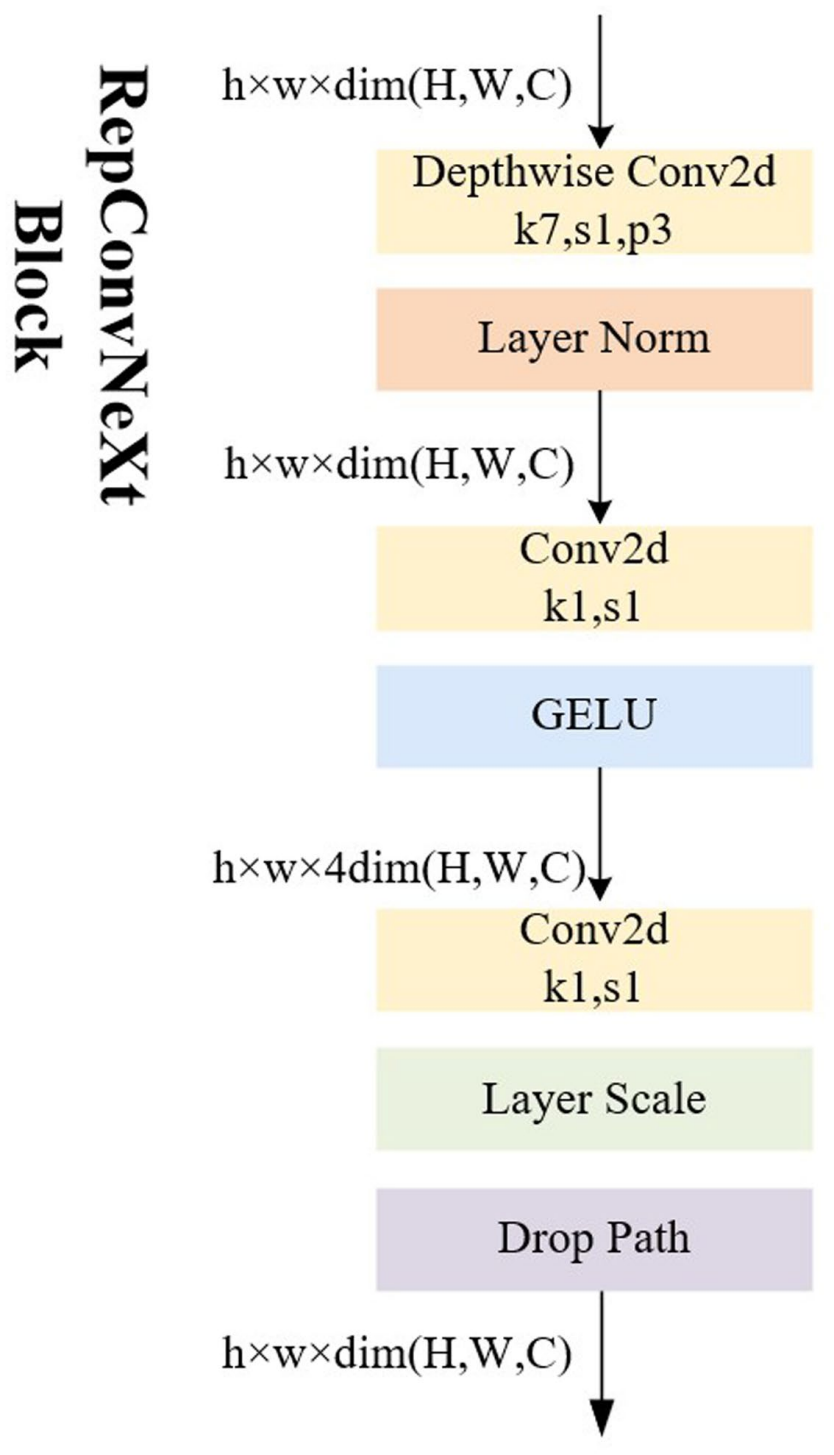
Illustrations of the architecture of RepConvNeXt Block.

The proposed module, transforms the ConvNeXt Block into a RepConvNeXt Block—a one-way structure resembling RepVgg (Ding et al., 2021)—during prediction process to further enhance real-time performance, as depicted in Figure 5. During training, using multi-branch structures such as ResNet or models like DenseNet (Huang et al., 2017) generally increases the model’s representational capacity by parallelizing multiple branches.

**Figure 6:**
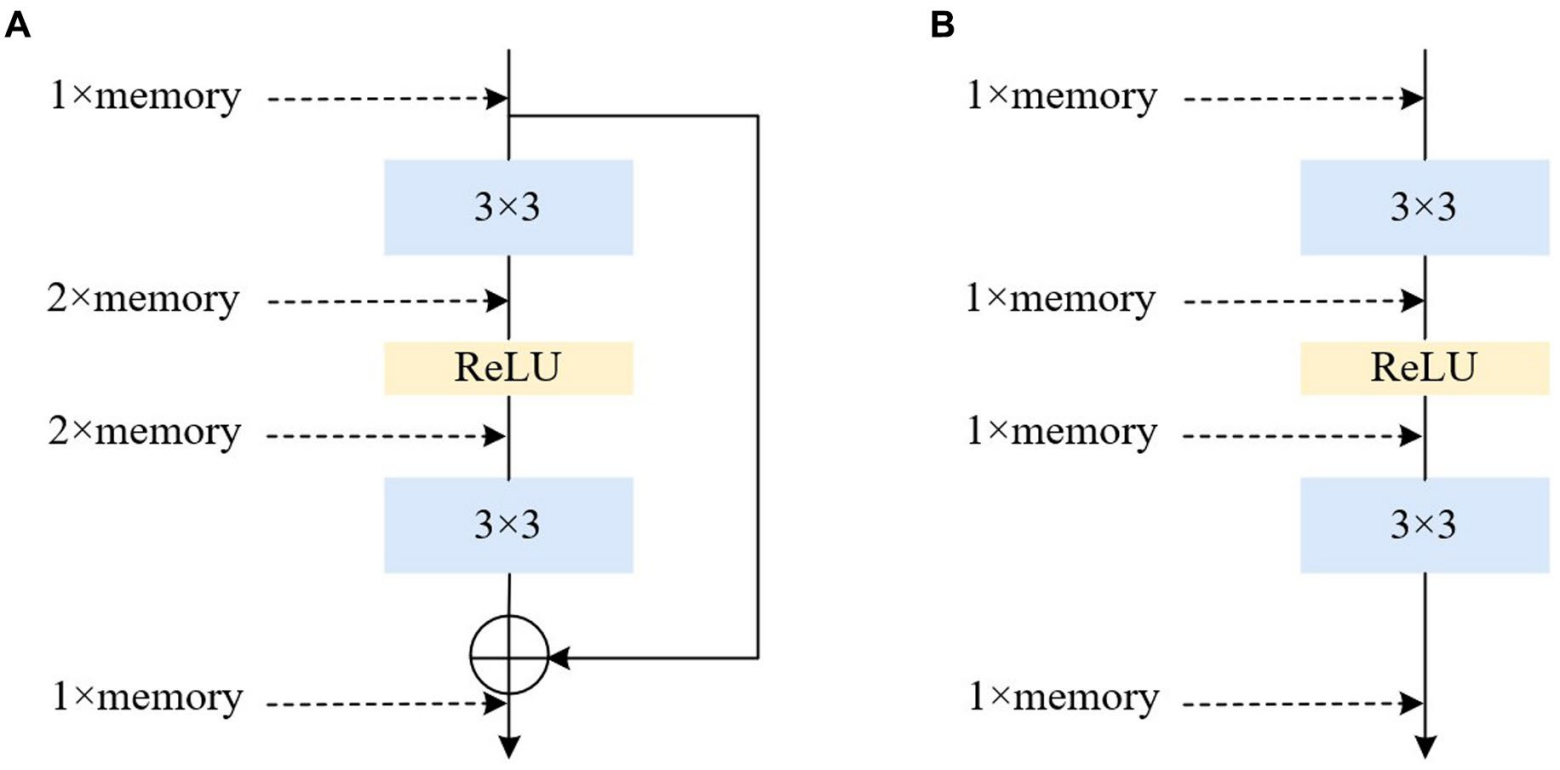
Illustrations of the architecture of multiple and single path. **(A)** Residual. **(B)** Plain.

Converting the multi-branch into a single-path topology during inference offers several advantages: Firstly, it enhances speed. Considering the degree of parallelism in hardware computation and MAC (memory access cost) during model reasoning, multi-branch models require separate computation of results for each branch. Some branches may compute faster while others compute more slowly, leading to potential underutilization of hardware arithmetic and insufficient parallelism. Additionally, each branch necessitates memory access and storage, resulting in substantial time wasted on IO operations. Secondly, it improves memory efficiency. The residual module depicted in Figure 6A, assuming the convolutional layer does not alter the number of channels, requires storing the respective feature maps on both the main branch and the shortcut branch, leading to roughly twice the memory consumption of the input activation before the add operation. Conversely, the structure shown in Figure 6B maintains the same memory usage throughout.

### 2.5. Classification

Through enhancements made to the Poolformer encoder, the output of the Transformer encoder after sequence pooling to the L-layer differs from the traditional ViT model. Instead of generating classification results by slicing the class token separately, the improved model utilizes data sequences containing both input image and class information. As a result, the model becomes more compact, and the sequence pooling output of the Transformer encoder produces sequential embedding in the latent space, enhancing the association with the input data. The final output obtained after sequence pooling can be utilized to derive results through a linear classifier.

## 3. Experiments and results

### 3.1. Dataset

**Figure 7:**
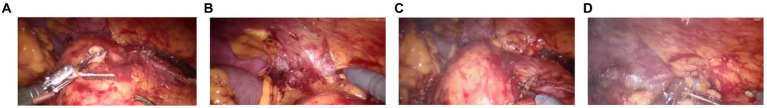
Experimental data set. **(A,B)** Original image. **(C,D)** Synthesized image with smoke.

For the experiment, real laparoscopic images from the Hamlyn Centre Laparoscopic/Endoscopic Video Dataset^1^ are employed, comprising 5,000 endoscopic images with dimensions of 384 × 192 pixels. As the images constitute a continuous video sequence with minimal differences between adjacent frames, to ensure the robustness of model training and the accuracy of model testing, we adopted a sampling approach. Specifically, we selected 5,000 images from the video dataset at irregular intervals and rendered 1,000 of them to generate a dataset comprising smoke-containing images, as illustrated in Figure 7. The remaining 4,000 images constitute the smoke-free dataset. The selected images is further partitioned into a training set (3,800 images) and a test set (1,200 images), maintaining a 4:1 ratio between smoke-free and smoke-containing images in each set. This balanced distribution ensures effective model training and evaluation.

This paper introduces Blender,^2^ a 3D graphic image engine, for software rendering to generate smoke-containing images, which enhances the neural network training dataset. The integration of software rendering addresses the limitation of smoke images in the real endoscopy image dataset. The Blender physical rendering engine is utilized to create realistic and accurate smoke textures, enabling the generation of simulated smoke with random shapes and densities. The rendered smoke possesses local color and transparency, with its position controlled by input parameters: random intensity (*T*_rand_), density (*D*_rand_), and position of smoke generation (*P*_rand_). The smoke image is defined as follow:


(1) $$I_{smoke´}\left(x,y\right)=\mathrm{Blender}\left(T_{\mathrm{rand}},D_{\mathrm{rand}},P_{\mathrm{rand}}\right)$$

The smoke image, denoted as *I_smoke_* (*x*, *y*), is synthesized by utilizing the luminance values of RGB channels. By fusing this rendered smoke with the laparoscopic image, the smoke-containing image is defined as follow:


(2) $$I_{simage}\left(x,y\right)=I_{original}\left(x,y\right)+I_{smoke}\left(x,y\right)$$

### 3.2. Experimental platforms

The experimental platform used in this study consists of a Windows 10 operating system, 8 GB RAM, a single NVIDIA 2080Ti 11 GB GPU, and a sixth-generation Intel^®^ Core™ i5 (4C4T) processor. CUDA 10.2, the computing platform provided by NVIDIA, is installed on this platform. The PyTorch 1.8.1 framework is employed to implement the endoscopic smoke image classification algorithm presented in this paper.

### 3.3. Experimental setup

In the training process of endoscopic smoke image classification, the hyperparameters for image training were set as follows: The dataset images were resized to a size of 224 × 224 using the transforms. Resize function as input to the Convolutional Tokenization layer. An exponential decay method was applied to adjust the learning rate, starting with an initial learning rate of 0.001. To enhance the number of Poolformer encoders and prevent overfitting, L = 10 was employed, and data augmentation was implemented through random level inversion. The training was conducted using a 10-fold cross-validation method with 50 epochs.

The experiments were conducted by the controlled variable method on endoscopic images for multiple separate groups, including the following network architectures: mobilenet_v3 (Howard et al., 2019), efficientnet_b7 (Tan and Le, 2019), the ViT network (ViT-B/16) (Dosovitskiy et al., 2021), Poolformer network with Token Mixer changed from attention to pooling layer (Poolformer_s12) (Yu et al., 2022), improved Poolformer network with the utilization of multiplexed branching structure akin to ConvNeXt during training, and improved Poolformer network with the utilization of multi-branch structure during training and single-path structure during prediction.

## 4. Results

### 4.1. Evaluation metrics

For the classification algorithm of endoscopic images based on Poolformer, which is adopted in this paper, the metrics used for evaluation include Accuracy (Acc), Sensitivity (Sens), and inference speed/ frames per second (fps).


(3) $$Acc=\frac{TP+TN}{TP+FN+FP+TN}$$

(4) $$Sens=\frac{TP}{TP+FN}$$

where TP represents the number of true positive samples (images with smoke correctly predicted as images with smoke), FP represents the number of false positive samples (smoke-free images incorrectly predicted as images with smoke), FN represents the number of false negative samples (images with smoke incorrectly predicted as smoke-free images), and TN represents the number of true negative samples (smoke-free images correctly predicted as smoke-free images).

### 4.2. Method comparison

To verify the effectiveness of the model, multiple sets of comparison experiments were conducted using the same endoscopic image dataset and smoke rendering scenarios, along with consistent settings for the remaining experimental parameters. The results were averaged over five runs, and the performance of different detection models on the dataset is presented in Table 1. Among the networks for comparison, all are lightweight neural networks designed for low-power devices, except for the classic ViT-B/16 network. The results reveal that in comparison to the mobilenet_v3, efficientnet_b7, and ViT-B/16 models, the proposed model demonstrates improvements in accuracy by 2, 1.6, and 1.4%, along with enhancements in sensitivity by 4.9, 3.2, and 2.7%, respectively. Furthermore, the proposed model achieves superior processing speed performance, with a frame rate increase of 30.9, 39.3, and 44.5 fps when compared to the mentioned models. These comparative experiments highlight the efficacy of the paper’s approach in conducting more accurate, comprehensive, and expeditious screening of smoke-containing images within endoscopic scenes, surpassing these existing modeling methodologies.

**Table 1:** The results of comparable experiments on different classification model.

Model	Acc/%	Sens/%	Inference Speed /fps
mobilenet_v3	93.9	78.6	56.2
efficientnet_b7	94.3	80.3	47.8
VIT-B/16	94.5	80.8	42.6
Our method	95.9	83.5	87.1

### 4.3. Ablation experiment

To evaluate the effectiveness of the improved multi-branch structure and the single-path inference process, we compare the performance of the original Poolformer model with versions that incorporate the multi-path structure alone and in combination with the single-path structure for real endoscopic image classification. The comparative experiments are presented in Table 2. The results demonstrate that the enhanced model, which incorporates a multiplexed branching structure, surpasses the original Poolformer model in terms of classification performance on the dataset. Specifically, the enhanced model exhibited a 2.8% enhancement in accuracy and a notable 9.6% increment in sensitivity. This outcome substantiates the efficacy of replacing the conventional pooling layer with a multiplexed branching structure within the Poolformer architecture, effectively bolstering detail retention within the endoscopic environment. However, the incorporation of this structure introduced a minor drawback, resulting in a reduction of processing speed by 26.3 fps. Further refinement of the model, encompassing a training process enriched with the multiplexed branching structure and a prediction network strengthened by a single-path topology, yielded commendable results. This adaptation yielded a 2.3% enhancement in accuracy and an 8.2% augmentation in sensitivity. Remarkably, this performance boost incurred only a marginal 6.4 fps decline in processing speed compared to the original Poolformer model. Thus, the strategic integration of the multiplexed branching structure into the training network emerged as a viable approach to amplify detail retention in the endoscopic environment. The incorporation of RepConvNeXt structure concurrently elevated processing speed, thereby enhancing endoscopic smoke classification performance and reducing processing time. Conclusively, the experimental results demonstrate the significant capability of the approach proposed in this study. This approach effectively enhances the detection prowess of the Poolformer model in the endoscopic image while concurrently sustaining its efficient real-time operational cadence.

**Table 2:** Ablations study for each component of our method.

Seq	Poolformer_s12	Multi-branch Structure	Single-path Structure	Acc/%	Sens/%	Inference Speed /fps
1	√			93.6	75.3	93.5
2	√	√		96.4	84.9	67.2
3	√	√	√	95.9	83.5	87.1

## 5. Conclusion

This paper introduces an improved Poolformer model for the automatic classification and recognition of endoscopic images containing smoke. The proposed model enhances the Token Mixer in the encoder by replacing the simple pooling layer with a multiplexed branching structure, similar to the pure convolutional neural network ConvNeXt. During the prediction process, the structure transforms into single-way, further improving the inference speed.

The experimental findings establish the superiority of our proposed method in the field of endoscopic image classification. In comparison to the traditional ViT-B16 network and the newer, lightweight networks including mobilenet_v3 and efficientnet_b7, our model exhibits substantial improvements. Specifically, it achieves an enhanced accuracy of 1.4, 2, and 1.6%, alongside sensitivity improvements of 2.7, 4.9, and 3.2%, respectively. Notably, these enhancements are accompanied by a significant boost in inference speed, with improvements of 44.5, 30.9, and 39.3 fps, respectively. These performance gains are attained without any appreciable degradation in image processing speed, underscoring the model’s efficiency. Furthermore, in contrast to the Poolformer framework, our model achieves these performance enhancements while maintaining image processing speeds, thus ensuring real-time processing remains unaffected. Comparatively, when compared to Poolformer_s12, our proposed method excels further, achieving an accuracy increase of 2.3% and a sensitivity boost of 8.2%. Although there is a marginal reduction in processing speed by 6.4 fps, these trade-offs emphasize the method’s prowess in smoke feature recognition and real-time processing efficiency within endoscopic environments. This method serves as an effective means for real-time screening of smoke-containing images in endoscopes, paving the way for potential integration with smoke removal techniques. Such integration can lead to more targeted and precise desmoking, avoiding the issues arising from the enhancing of smoke-free images, notably mitigating computational overhead. By introducing real-time smoke detection into endoscopic procedures, we aspire to reduce equipment resource requirements, augment processing speed, and enhance the real-time, precision, and scientific validity of smoke removal in endoscopic settings.

## Data availability statement

The original contributions presented in the study are included in the article/supplementary material, further inquiries can be directed to the corresponding author.

## Author contributions

HW: Conceptualization, Data curation, Funding acquisition, Methodology, Project administration, Writing – original draft, Writing – review & editing. KW: Conceptualization, Data curation, Methodology, Writing – original draft, Software, Validation, Writing – review & editing. TY: Conceptualization, Data curation, Methodology, Validation, Writing – review & editing. HZ: Conceptualization, Data curation, Methodology, Writing – review & editing. EC: Conceptualization, Data curation, Methodology, Writing – review & editing. YL: Conceptualization, Funding acquisition, Investigation, Methodology, Writing – review & editing. YW: Conceptualization, Funding acquisition, Methodology, Writing – review & editing. JL: Conceptualization, Methodology, Writing – review & editing, Funding acquisition. YP: Conceptualization, Funding acquisition, Methodology, Writing – review & editing.

## Funding

The author(s) declare financial support was received for the research, authorship, and/or publication of this article. The project was funded by the Science and Technology Research Program of Chongqing Municipal Education Commission under Grant No. KJQN202100602, KJQN202300637, KJQN202300613 and KJQN202000604; Chongqing Technical Innovation and Application Development Special Project under Grant No. CSTB2022TIAD-KPX0062 and cstc2021jscx-gksbx0051; Project funded by China Postdoctoral Science Foundation under Grant No. 2022MD713702, Special Postdoctoral Support from Chongqing Municipal People’s Social Security Bureau under Grant No. 2021XM3010; Nature Science Foundation of Chongqing under Grant No. CSTC2021JCYJ-BSH0221, 2022NSCQ-LZX0254 and CSTB2022NSCQ-MSX1523, National Natural Science Foundation of China under Grant No. U21A20447 and 61971079, Chongqing Innovation Group Project under Grant No. cstc2020jcyj- cxttX0002.

## Conflict of interest

HW and YW were employed by Chongqing Xishan Science & Technology Co., Ltd.

The remaining authors declare that the research was conducted in the absence of any commercial or financial relationships that could be construed as a potential conflict of interest.

## Publisher’s note

All claims expressed in this article are solely those of the authors and do not necessarily represent those of their affiliated organizations, or those of the publisher, the editors and the reviewers. Any product that may be evaluated in this article, or claim that may be made by its manufacturer, is not guaranteed or endorsed by the publisher.

## Footnotes

^1^ http://hamlyn.doc.ic.ac.uk/vision/

^2^ https://www.blender.org/
